# Glucocorticoids Suppress Antimicrobial Autophagy and Nitric Oxide Production and Facilitate Mycobacterial Survival in Macrophages

**DOI:** 10.1038/s41598-017-01174-9

**Published:** 2017-04-20

**Authors:** Jinli Wang, Ruining Wang, Hui Wang, Xiaofan Yang, Jiahui Yang, Wenjing Xiong, Qian Wen, Li Ma

**Affiliations:** Institute of Molecular Immunology, School of Laboratory Medicine and Biotechnology, Southern Medical University, Guangzhou, 510515 China

## Abstract

Chronic administration of glucocorticoids has been shown to render individuals highly susceptible to mycobacterial infection and lead to reactivation of latent bacilli. However, the effect of glucocorticoids on innate anti-mycobacterial defense, especially in macrophages remains largely unknown. Here, we found that glucocorticoids inhibited the innate immune response, antimicrobial nitric oxide production and autophagy in mycobacteria-challenged macrophages. Meanwhile, maturation and acidification of mycobacterial phagosomes were attenuated in RAW264.7 cells after glucocorticoids treatment. Consequently, we observed a glucocorticoid-induced increase in the survival of intracellular mycobacteria in both primary macrophages and cell lines. Glucocorticoids treatment decreased the activation of TBK1 kinase, which promotes the maturation of autophagosomes. Inhibition of TBK1 also decreased the production of nitric oxide. Furthermore, several autophagy-related genes were down-regulated, while activation of the Akt/mTOR signaling pathway was increased after glucocorticoids treatment, which may account for autophagy inhibition during mycobacterial infection. Restoration of autophagy with the agonist rapamycin abolished glucocorticoid-mediated enhancement of mycobacterial survival, suggesting that glucocorticoids blocked anti-mycobacterial defense via autophagy inhibition. Collectively, this study demonstrates that glucocorticoids impair innate antimicrobial autophagy and promote mycobacterial survival in macrophages, which is a novel mechanism for glucocorticoid-mediated immunosuppression. Our findings may provide important clues for tuberculosis prevention.

## Introduction

Tuberculosis (TB) remains a major global health challenge and is the leading cause of mortality among infectious diseases worldwide, with approximately 1.5 million deaths annually^1^. *Mycobacterium tuberculosis* (MTB), the causative agent of TB, infects approximately one-third of the global population. Overall, a relatively small proportion (5–15%) of the estimated 2–3 billion infected individuals will develop active TB disease during their lifetime, while the remaining undergo asymptomatic latent infection^2^. This fact highlights the importance of host immunity in controlling MTB infection.

Several extrinsic and intrinsic factors may impair the immune system and render individuals susceptible to MTB infection or result in reactivation of latent MTB. For example, human immunodeficiency virus (HIV) infection impairs host CD4^+^ cell response, which leads to secondary infection with MTB and exacerbates the latter disease^3^. An inheritable deficiency in ubiquitin-like intracellular protein interferon stimulated gene (ISG)-15 reduces the production of interferon (IFN)-γ by lymphocytes and significantly enhances susceptibility to mycobacterial disease in humans^4^. Additionally, several iatrogenic factors such as the widely-used immunosuppressive agent glucocorticoids, may also disrupt host anti-mycobacterial defense. Therefore, it is crucial to identify risk factors for TB and elucidate the underlying mechanisms for effective prevention of TB reactivation in the future.

Glucocorticoids are steroid hormones that control a variety of fundamental metabolic and homeostatic functions. Synthetic glucocorticoids, such as dexamethasone and hydrocortisone, are generally prescribed in clinics to treat autoimmune and inflammatory diseases, such as rheumatoid arthritis, ulcerative colitis and systemic lupus erythematosus. However, clinical observations have shown that patients treated with glucocorticoids have a substantially increased risk of developing TB^5–7^. In a TB animal model, glucocorticoids treatment after containment resulted in reactivation of the disease^8^. Several previous reports have demonstrated that glucocorticoids inhibited the proliferation of antigen-specific T cells^9^. An increased rate of apoptosis and a decrease in IFN-γ secretion were observed in cultured T cells after glucocorticoid methylprednisolone treatment^10^. In addition, during helper T cell (Th) polarization, glucocorticoids may cause a shift in the Th1/Th2 balance toward a Th2 dominant response, which is detrimental to TB control^11, 12^. Nevertheless, whether glucocorticoids modulate another arm of the immune system, innate immune defense against mycobacterial infection, remains largely unknown.

Macrophages are major innate immune cells; they are invaded by MTB, which resides in these cells. The invading bacilli are sensed by pattern recognition receptors (PRRs), which initiate the host innate immune response in macrophages^13^. Pro-inflammatory cytokines and chemokines are secreted at the infection site to recruit different types of leukocytes and orchestrate immune responses and host anti-mycobacterial defense. Several mechanisms are deployed by macrophages to combat invading MTB, such as nitric oxide (NO) and antimicrobial peptides^14^. In addition, numerous studies in the last decade have demonstrated that the autophagy pathway is activated via PRR signaling or other immunological stimuli, such as T cell-derived IFN-γ, to exert antimicrobial effects^15, 16^.

Autophagy is an evolutionarily conserved biological process, which is triggered under starvation circumstances by sequential activation of a range of autophagy-related genes (ATGs), such as ATG5, ATG6, ATG7 and ATG12^17^. Intracellular aggregated proteins and damaged mitochondria are degraded via the autophagy pathway to maintain cytoplasmic homeostasis. Importantly, recent reports have established the critical role of autophagy in antimicrobial defense against intracellular pathogens, such as MTB^15, 18^. While MTB escapes the host defense by inhibiting phagosome maturation, autophagy promotes the fusion of the MTB phagosome with autophagosomes and facilitates subsequent clearance of the bacilli in autophagolysosomes^19, 20^. Additionally, antigen presentation capability is enhanced by autophagy in macrophages to elicit protective adaptive immune response to mycobacteria. Deficiency in key ATGs, such as ATG5 in myeloid cells, renders mice highly susceptible to MTB infection with a substantially increased bacterial burden in the lungs^21^.

The autophagic process is modulated by multiple signaling pathways. Mammalian target of rapamycin (mTOR), a highly conserved serine/threonine kinase, serves as a master negative regulator of autophagy induction^22^. mTOR negatively regulates autophagy by inhibiting the function of the unc-51-like kinase 1 (ULK1)/ULK2 complex, which plays an important role in autophagy initiation^22^. mTOR is inhibited by the immunosuppressant rapamycin, which is therefore generally used as an autophagy agonist. mTOR activation is regulated downstream of the class I PI3K- and v-akt murine thymoma viral oncogene homolog (Akt)/protein kinase B (PKB) pathway, and activation of the PI3K-Akt pathway activates mTOR, resulting in suppression of autophagy^22^. In addition, TANK-binding kinase 1 (TBK1) coordinates assembly and function of the autophagic machinery by phosphorylating the autophagic adaptor p62 (sequestosome1), thus promoting the maturation of autophagosomes and the elimination of intracellular mycobacteria^23^.

Here, we investigated the role of glucocorticoids in innate host defense against mycobacterial infection in macrophages. Critical anti-mycobacterial machinery—NO and autophagy—were inhibited in macrophages after glucocorticoid treatment. Simultaneously, we observed impaired maturation of mycobacterial phagosomes in glucocorticoid-treated macrophages and an increase in the survival of intracellular mycobacteria. Furthermore, we found that the activation of TBK1 and key ATGs expression were down-regulated, while the activation of the Akt/mTOR signaling pathway was enhanced in glucocorticoid-treated macrophages, thus hindering autophagic flux. Our findings provide new insight into the mechanism of the glucocorticoid-mediated immunosuppression and TB susceptibility, which has important implications for the prevention of TB reactivation.

## Results

### Glucocorticoids inhibit the innate immune response, nitric oxide production and autophagy in macrophages after mycobacterial infection

We sought to demonstrate the effect of glucocorticoids on innate immunity during mycobacterial infection. First, we treated mycobacteria-challenged macrophages with the synthetic glucocorticoid dexamethasone and analyzed inflammatory cytokines with real-time PCR. *M. bovis* BCG-induced levels of IL-1β, IL-6 and TNFα were substantially decreased in dexamethasone-treated macrophage-like RAW264.7 cells (Fig. S1a, all *p* < 0.001). Similarly, hydrocortisone, a short-term glucocorticoid strongly reduced all inflammatory cytokines tested (Fig. S1b, all *p* < 0.001). Moreover, *M. tuberculosis* H37Rv-induced levels of IL-1β, IL-6 and TNFα were also decreased dramatically in dexamethasone-treated RAW264.7 cells (Fig. S1c), indicating glucocorticoid-mediated inhibition of innate immunity after mycobacterial infection.

**Figure 1:**
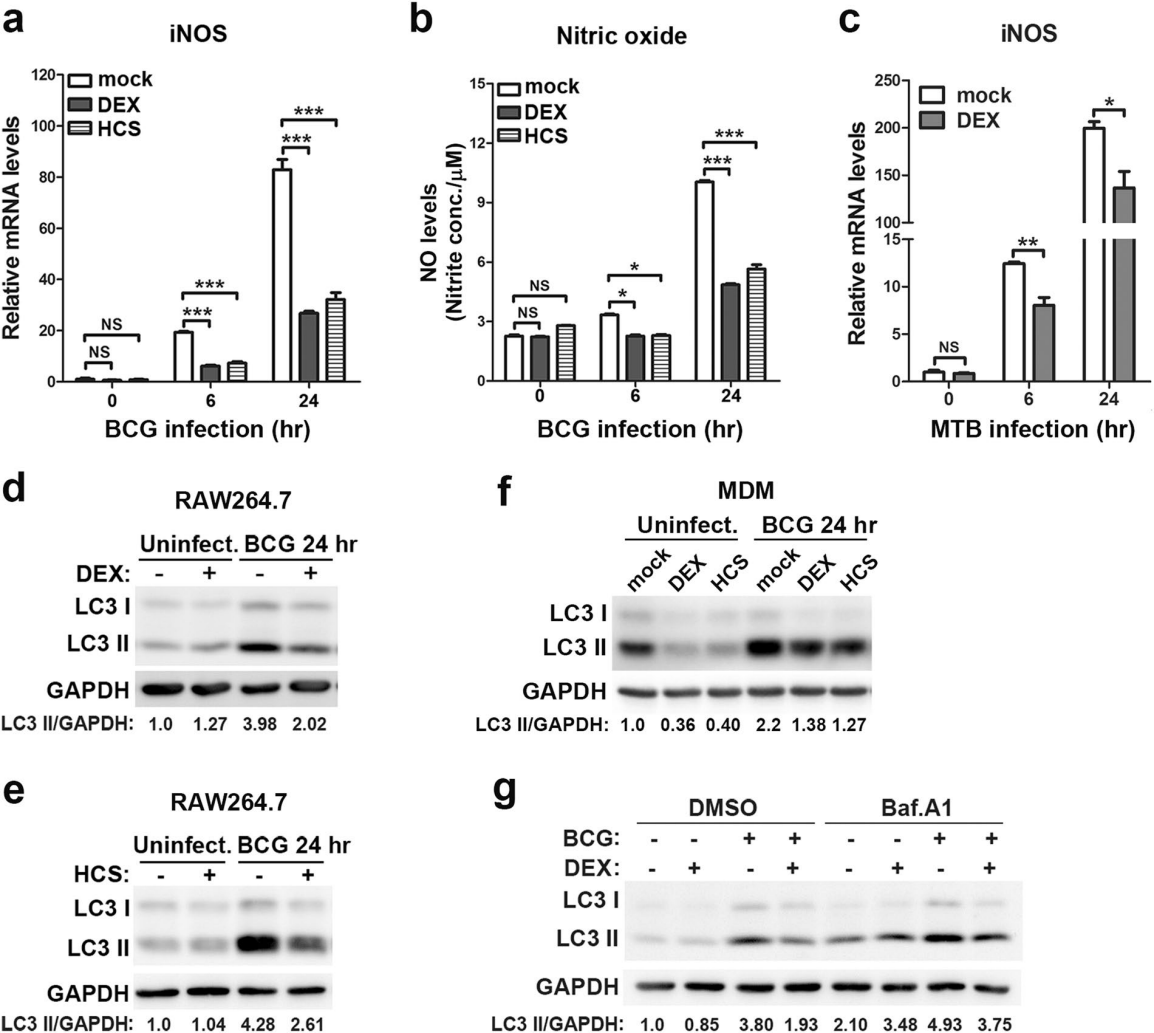
Glucocorticoids suppress iNOS/NO and autophagy in mycobacteria-challenged macrophages. (**a**,**b**) RAW264.7 cells were pretreated with dexamethasone (DEX) (1 μM), hydrocortisone (HCS) (1 μM) or vehicle control ethanol (mock) for 6 hr and were then challenged with *M. bovis* BCG (MOI 5) for the indicated time. iNOS mRNA level was assessed with real-time PCR (**a**). NO in the supernatant was detected with the Griess assay (**b**). (**c**) RAW264.7 cells were pretreated with DEX (1 μM) or vehicle control ethanol (mock) for 6 hr and then challenged with *M. tuberculosis* H37Rv (MOI 5) for the indicated time. iNOS mRNA level was assessed with real-time PCR. (**d**,**e**) RAW264.7 cells were pretreated with DEX (1 μM) (**d**), HCS (1 μM) (**e**) or vehicle control ethanol (mock) for 6 hr and then challenged with *M. bovis* BCG (MOI 5) for 24 hr. The LC3 level was detected with Western blot analysis. Full-length blots are presented in Supplementary Figs 4 and 5, respectively. (**f**) MDMs were pretreated with DEX (1 μM), HCS (1 μM) or vehicle control ethanol (mock) for 6 hr and then challenged with *M. bovis* BCG (MOI 5) for 24 hr. The LC3 level was detected with Western blot analysis. Full-length blots are presented in Supplementary Fig. 6. (**g**) RAW264.7 cells were treated as described in (**d**), and cells were treated with Bafilomycin A1 (Baf. A1, 100 nM) 2 hr before cellular protein extraction for Western blot analysis. The LC3 level was detected with Western blot analysis. Full-length blots are presented in Supplementary Fig. 7. Relative band intensity of LC3 II was calculated after normalization by load control GAPDH and is shown below each lane. For Western blot analysis, GAPDH was used as the loading control, and data are representative of three independent experiments with similar results. For real-time PCR, the level of each mRNA was normalized to that of β-actin mRNA and is expressed relative to expression in unstimulated cells. Data are shown as the mean ± SEM of three independent experiments. *p < 0.05; **p < 0.01; ***p < 0.001. NS, not significant.

The induction of inducible NO synthase (iNOS) and release of NO were shown to be a powerful innate anti-mycobacterial defense mechanism in mice^24^. To determine whether glucocorticoids modulate the iNOS/NO pathway after mycobacterial infection in macrophages, we pretreated RAW264.7 cells with glucocorticoids and then infected the cells with *M. bovis* BCG. The expression of iNOS and the production of NO were detected. Our results showed that macrophages treated with either dexamethasone or hydrocortisone had significantly decreased iNOS expression (Fig. 1a) and NO production (Fig. 1b) after *M. bovis* BCG infection. Similarly, iNOS expression was also decreased after dexamethasone treatment in macrophages during *M. tuberculosis* H37Rv infection (Fig. 1c).

Recently autophagy has been recognized as an important component of innate immunity and has been shown as a potent antimicrobial mechanism^18^. We next explore whether glucocorticoids modulate antimicrobial autophagy after mycobacterial infection in macrophages. Induction of autophagy is characterized by lipidation of LC3 I to form LC3 II which binds to the membrane of autophagosomes. Western blot analysis revealed that mycobacterial infection up-regulated level of LC3 II in macrophages; however, dexamethasone treatment attenuated the increase of LC3 II, suggesting inhibition of autophagy (Fig. 1d). Hydrocortisone also displayed inhibitory effects on autophagy in mycobacteria-challenged macrophages (Fig. 1e). Furthermore, the effects of glucocorticoids on autophagy were also tested in human monocyte-derived macrophages (MDMs). Our results showed that MDMs pretreated with either dexamethasone or hydrocortisone showed significantly decreased the amount of LC3 II with or without *M. bovis* BCG infection (Fig. 1f). To assess the dynamic autophagic flux, we treated RAW264.7 cells with a selective vacuolar H^+^ ATPases inhibitor, Bafilomycin A1, to block cargo degradation in autolysosomes. In the presence of Bafilomycin A1, dexamethasone also suppressed the accumulation of LC3 II after mycobacterial infection (Fig. 1g), suggesting that glucocorticoids inhibited autophagy induction rather than promoting autophagosome-lysosome fusion.

**Figure 2:**
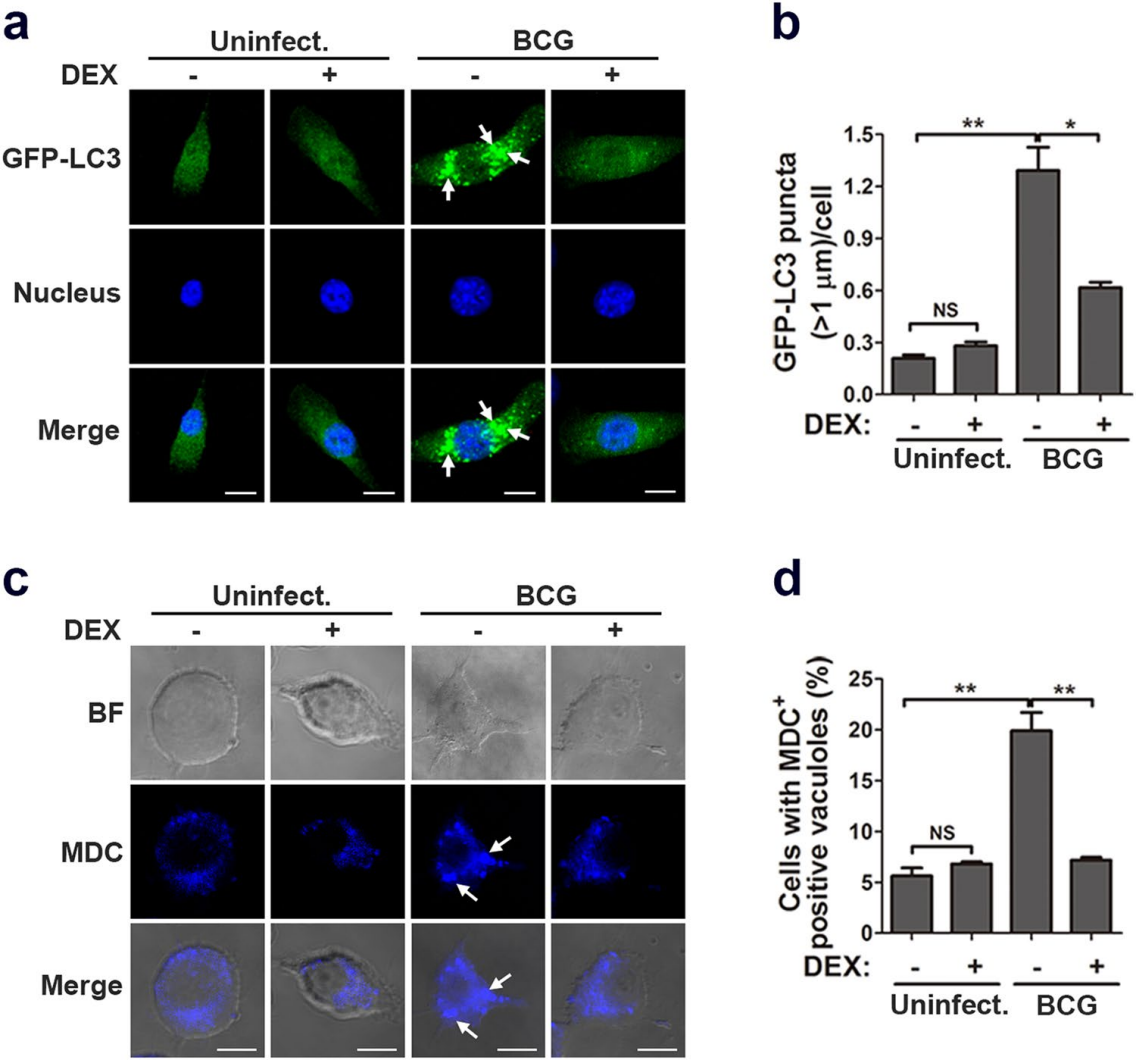
Glucocorticoids decrease autophagosome formation in mycobacteria-challenged macrophages. (**a**,**b**) RAW264.7 cells stably expressing GFP-LC3 were pretreated with dexamethasone (DEX) (1 μM) or vehicle control ethanol (mock) for 6 hr and then challenged with *M. bovis* BCG (MOI 5) for 24 hr. Nuclei were stained with DAPI (blue), and GFP-LC3 puncta were monitored with fluorescence microscopy (**a**). The average number of GFP-LC3 puncta (>1 μm) per cell was counted in at least 200 cells (**b**). (**c**,**d**) RAW264.7 cells were pretreated with DEX (1 μM) or vehicle control ethanol (mock) for 6 hr and then challenged with *M. bovis* BCG (MOI 5) for 24 hr. Autophagosomes were detected with MDC staining (**c**). The percentage of MDC vacuole-positive cells was calculated in at least 200 cells (**d**). Arrows indicate the GFP-LC3 puncta or MDC-positive vacuoles. Scale bar, 5 μm. *p < 0.05; **p < 0.01. NS, not significant.

Moreover, we examined the effect of glucocorticoids on autophagy by monitoring LC3 distribution in macrophages. RAW264.7 cells stably expressing GFP-LC3 were infected with *M. bovis* BCG following dexamethasone treatment. Fluorescence microscopy showed that BCG infection triggered obvious GFP-LC3 puncta in macrophages. However, dexamethasone significantly reduced the amount of GFP-LC3 puncta (Fig. 2a,b). Monodansylcadaverine (MDC) was used to label autophagosomes. The number of MDC positive autophagosomes was dramatically decreased in dexamethasone-treated macrophages after mycobacterial infection (Fig. 2c,d). Taken together, these data indicated that glucocorticoids hindered mycobacteria-induced autophagy in macrophages.

### Impaired maturation of mycobacterial phagosomes in macrophages after glucocorticoid treatment

**Figure 3:**
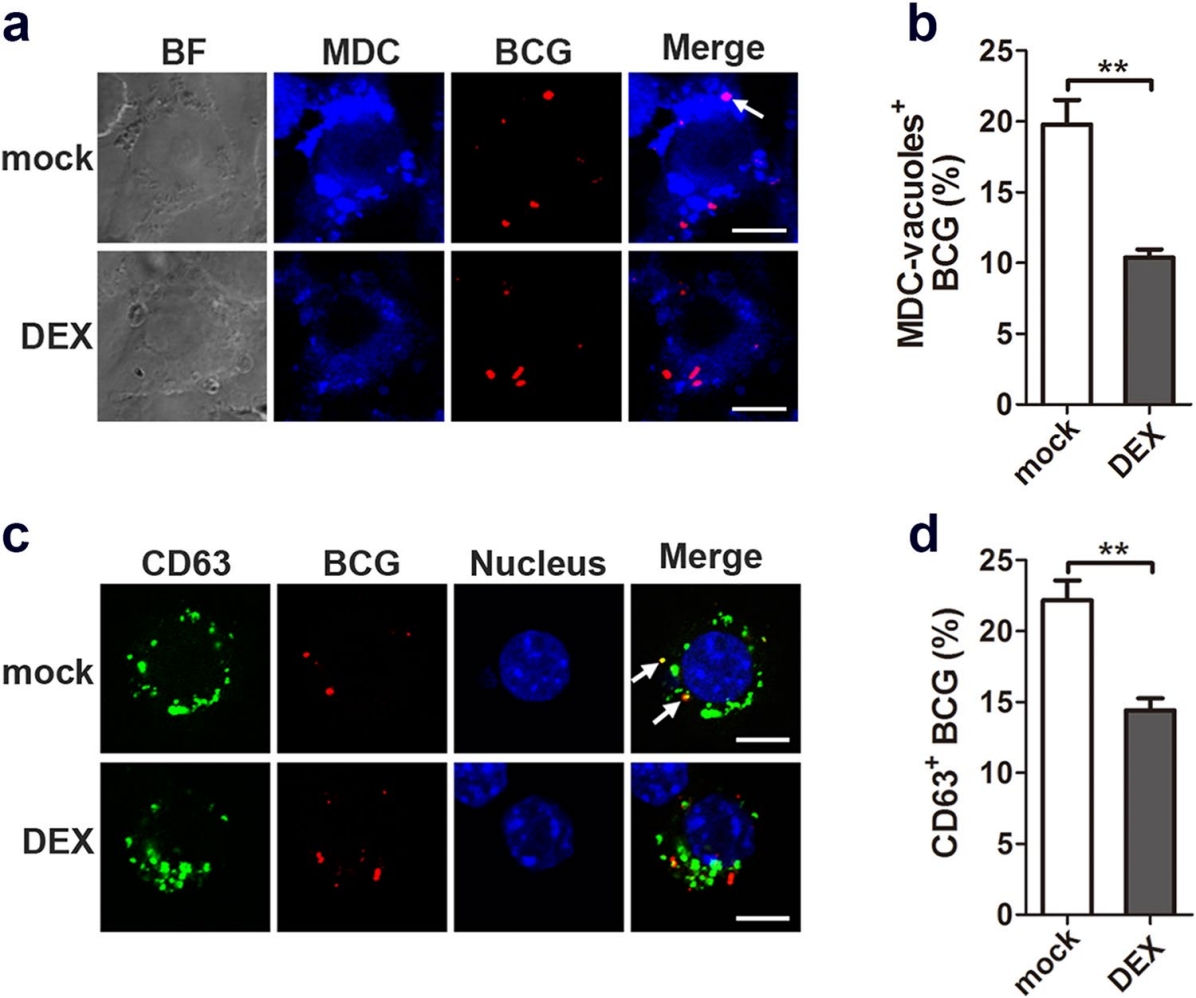
Glucocorticoids inhibit the maturation of BCG phagosomes in macrophages. (**a**,**b**) RAW264.7 cells were pretreated with dexamethasone (DEX) (1 μM) or vehicle control ethanol for 24 hr and then challenged with Texas-Red-labeled *M. bovis* BCG (MOI 10) for 1 hr. Autophagosomes were detected with MDC staining (**a**). The percentage of BCG phagosome colocalizing with MDC-positive vacuoles was calculated in at least 100 phagosomes (**b**). (**c**,**d**) RAW264.7 cells were treated as described in (**a**,**b**), and lysosomes were labeled with CD63 (green) (**c**). The percentage of BCG phagosome colocalizing with CD63-positive vacuoles was calculated in at least 100 phagosomes. (**d**). Scale bar, 5 μm. **p < 0.01.

Autophagy has been shown to deliver mycobacterial phagosomes to lysosomes, which overcomes phagosome maturation arrest and facilitates its acidification and subsequent clearance of intracellular bacilli^18^. Therefore, we next determined whether glucocorticoids modulate the maturation of mycobacterial phagosomes in macrophages. RAW264.7 cells were stained with MDC to display acidic vacuoles in the cytoplasm. Fluorescence microscopy revealed colocalization of MDC-positive vacuoles with Texas-Red-labeled BCG phagosomes in macrophages; however, dexamethasone treatment blocked their colocalization (Fig. 3a), with an approximately one half decrease in MDC-positive BCG phagosomes (Fig. 3b). Moreover, we monitored lysosomes with the specific marker CD63 and assessed the maturation of mycobacterial phagosomes. Dexamethasone-treated RAW264.7 cells showed reduced colocalization of lysosomes and BCG phagosomes (Fig. 3c,d), suggesting inhibition of mycobacterial phagosome maturation. Together, these data indicated that glucocorticoids impaired maturation of mycobacterial phagosomes in macrophages.

### Glucocorticoids suppress the clearance of intracellular mycobacteria in macrophages

**Figure 4:**
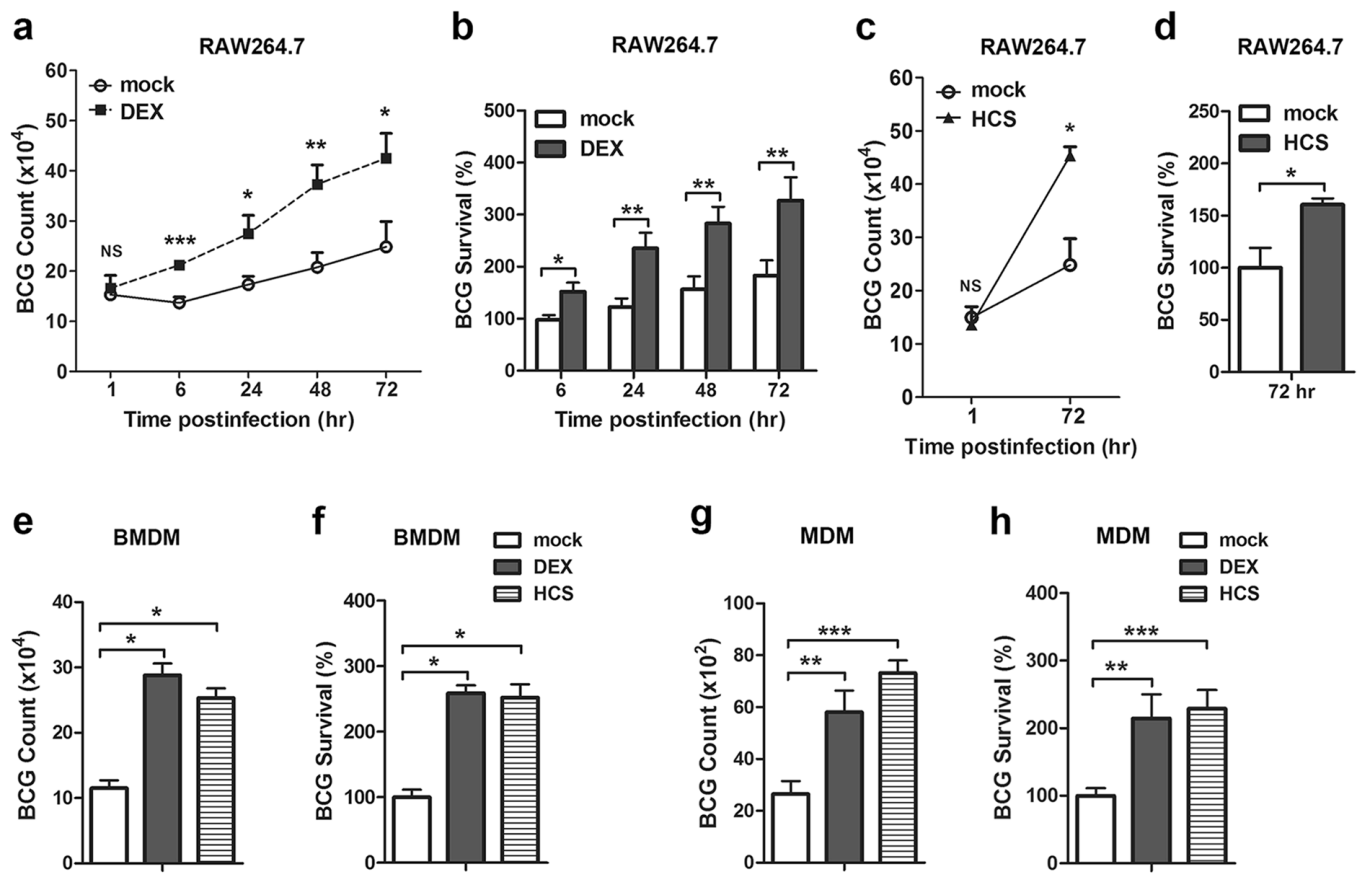
Increased mycobacterial survival in macrophages after glucocorticoids treatment. (**a**–**d**) RAW264.7 cells were pretreated with dexamethasone (DEX) (1 μM), hydrocortisone (HCS) (1 μM) or vehicle control ethanol for 24 hr and then challenged with *M. bovis* BCG (MOI 10) for 1 hr. The intracellular viable bacilli were determined by CFU assays at the indicated time post-infection (**a**,**c**). Survival rate was calculated compared with that of mock-treated cells (**b**,**d**). (**e–h**) BMDMs (**e**,**f**) and MDMs (**g**,**h**) were treated like RAW264.7 cells as described in (**a–d**). The intracellular viable bacilli were determined by CFU assays at 72 hpi (**e**,**g**). Survival rate was calculated compared with that of the mock-treated cells (**f**,**h**). Data are shown as the mean ± SEM of three independent experiments. *p < 0.05; **p < 0.01; ***p < 0.001. NS, not significant.

The glucocorticoid-mediated suppression of antimicrobial autophagy and mycobacterial phagosome maturation prompted us to further determine their role in clearance of intracellular mycobacteria. RAW264.7 cells were pretreated with dexamethasone followed by *M. bovis* BCG infection, and intracellular viable bacteria were detected with colony-forming unit (CFU) assays. We found that dexamethasone increased the survival of mycobacteria in macrophages at 6, 24, 48, 72 hr post-infection (hpi) (Fig. 4a,b). Intracellular viable bacilli in dexamethasone-treated RAW264.7 cells at 72 hpi were approximately 2-fold greater than those in mock-treated cells (Fig. 4a). Moreover, the percentage of viable mycobacteria, which was calculated compared with the initial internalized bacilli in macrophages, also showed increased mycobacterial survival in dexamethasone-treated cells (Fig. 4b). Cell viability was measured by propidium iodide (PI) staining after pretreatment with dexamethasone and infection with *M. bovis* BCG for the indicated times (Fig. S2). Our results showed no major decrease in cells viability of either vehicle or dexamethasone treatment through 72 hpi (Fig. S2). To confirm the effects of glucocorticoids on mycobacterial survival, we used another glucocorticoid, hydrocortisone, to treated RAW264.7 cells. Similarly, we observed a substantial increase in viable bacilli after hydrocortisone treatment (Fig. 4c,d). We then determined the role of glucocorticoids in mycobacterial survival using primary bone-marrow-derived macrophages (BMDMs), in which both glucocorticoid agents used promoted the survival of mycobacteria (Fig. 4e,f). MDMs pretreated with either dexamethasone or hydrocortisone showed an increase in the survival of intracellular mycobacteria (Fig. 4g,h). Collectively, these results implied that glucocorticoids antagonized the innate antimicrobial defense against mycobacteria in macrophages.

To further assess whether glucocorticoids affect macrophage-mediated phagocytosis of *M. bovis* BCG, we pretreated RAW264.7 cells with glucocorticoid agents, followed by fluorescent Texas-Red-labeled *M. bovis* BCG infection, and phagocytosis was analyzed by flow cytometry. Our results showed that neither dexamethasone nor hydrocortisone had major effects on *M. bovis* BCG phagocytosis by macrophages (Fig. S3a,b). Moreover, the uptake of *M. bovis* BCG in macrophages was detected by CFU assays after glucocorticoid treatment. Similarly, RAW264.7 cells pretreated with glucocorticoids showed no difference in the phagocytosis of *M. bovis* BCG compared with that of mock-treated cells (Fig. S3c,d). The above results indicated that glucocorticoids inhibited intracellular killing of mycobacteria in macrophages without affecting mycobacterial phagocytosis.

### Glucocorticoids suppress TBK1 activation in mycobacteria-challenged macrophages

**Figure 5:**
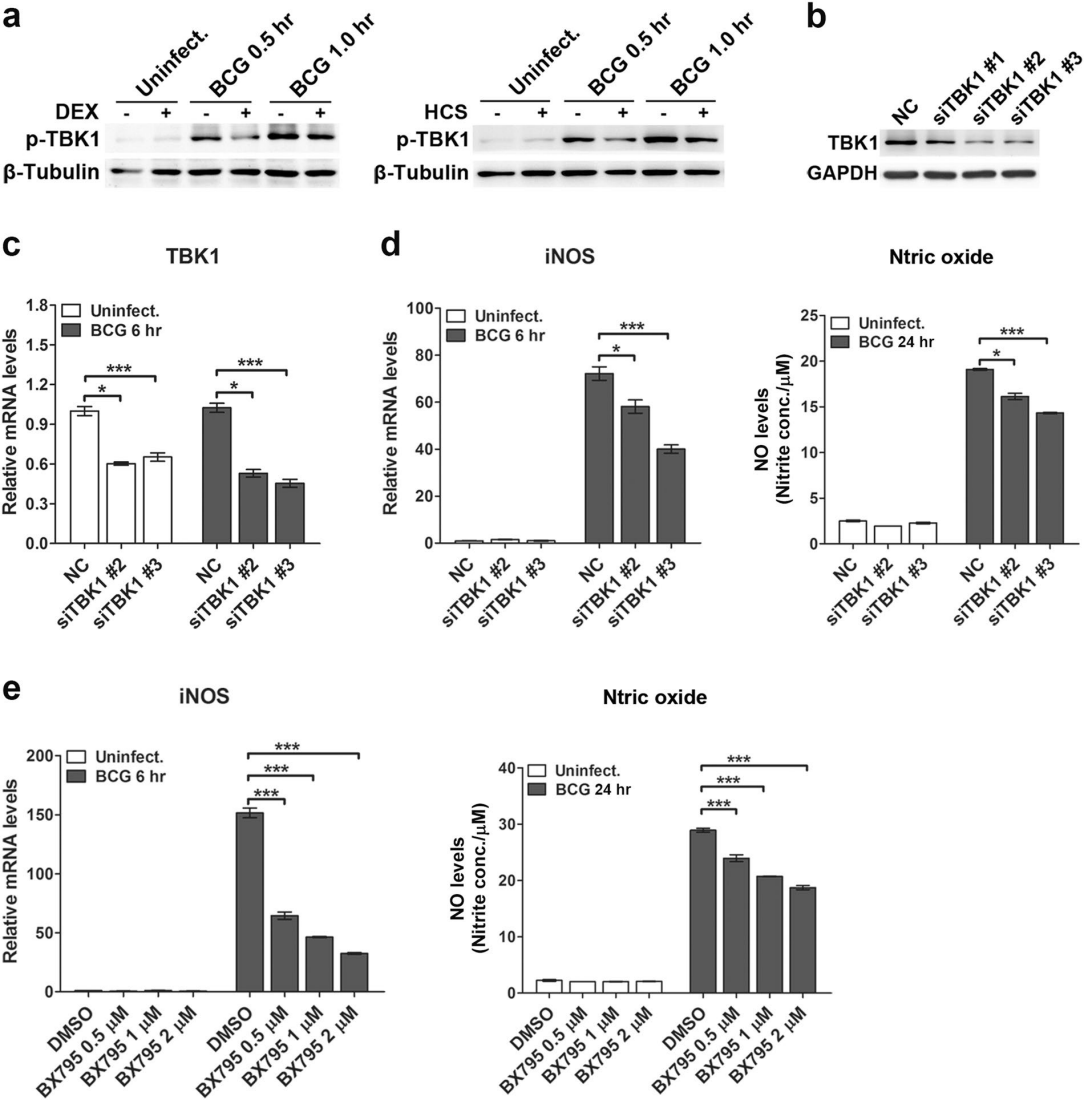
Glucocorticoids inhibit TBK1 activation, and TBK1 silencing decreases iNOS expression and NO production in macrophages during mycobacterial infection. (**a**) RAW264.7 cells were pretreated with dexamethasone (DEX) (1 μM), hydrocortisone (HCS) (1 μM) or vehicle control ethanol (mock) for 24 hr and then challenged with *M. bovis* BCG (MOI 5) for the indicated times. Then the phosphorylation level of TBK1 was tested with Western blot analysis. Full-length blots are presented in Supplementary Fig. 8. (**b**) RAW264.7 cells were transfected with control siRNA (NC) or three TBK1 siRNAs (siTBK1 #1, #2, #3) with different sequences for 24 hr, and the protein level of TBK1 was tested with Western blot. Full-length blots are presented in Supplementary Fig. 9. (**c**,**d**) RAW264.7 cells were transfected with NC or siTBK1 #2 or #3 for 24 hr, and then infected with *M. bovis* BCG for the indicated times. TBK1 (**c**) and iNOS (**d**) mRNA level were tested with real-time PCR. NO in the supernatant was detected with the Griess assay (**d**). (**e**) RAW264.7 cells were pretreated with a TBK1 inhibitor (BX795) at the indicated concentration for 1 hr, and then infected with *M. bovis* BCG for the indicated times. iNOS mRNA levels were tested with real-time PCR. NO in the supernatant was detected with the Griess assay. For Western blot analysis, β-Tubulin or GAPDH was used as a loading control, and data are representative of three independent experiments with similar results. For real-time PCR, the level of each mRNA was normalized to that of β-actin mRNA and is expressed relative to expression in unstimulated cells. Data are shown as the mean ± SEM of three independent experiments. *p < 0.05; ***p < 0.001.

In the autophagy-mediated anti-mycobacterial response, TBK1 is reported to be essential for the maturation of autophagosomes^23^. Therefore, we next explored whether glucocorticoids modulate TBK1 activation after mycobacterial infection in macrophages. Both dexamethasone and hydrocortisone decreased phosphorylated TBK1 in RAW264.7 cells after *M. bovis* BCG infection (Fig. 5a). To further determine whether inhibition of TBK1 affected the iNOS/NO pathway, we knocked down TBK1 by RNA interference. Western blot analysis showed that siRNA #2 and siRNA #3 had a stronger silencing efficiency than that of siRNA #1 (Fig. 5b,c). TBK1-silenced cells had attenuated iNOS expression and NO production (Fig. 5d). Moreover, the TBK1 specific inhibitor BX795 was used to inhibit its activity. Cells treated with BX795 showed significantly decreased iNOS expression and NO production after mycobacterial infection (Fig. 5e).

### Glucocorticoids repress ATG expression and enhance Akt/mTOR pathway activation in mycobacteria-challenged macrophages

**Figure 6:**
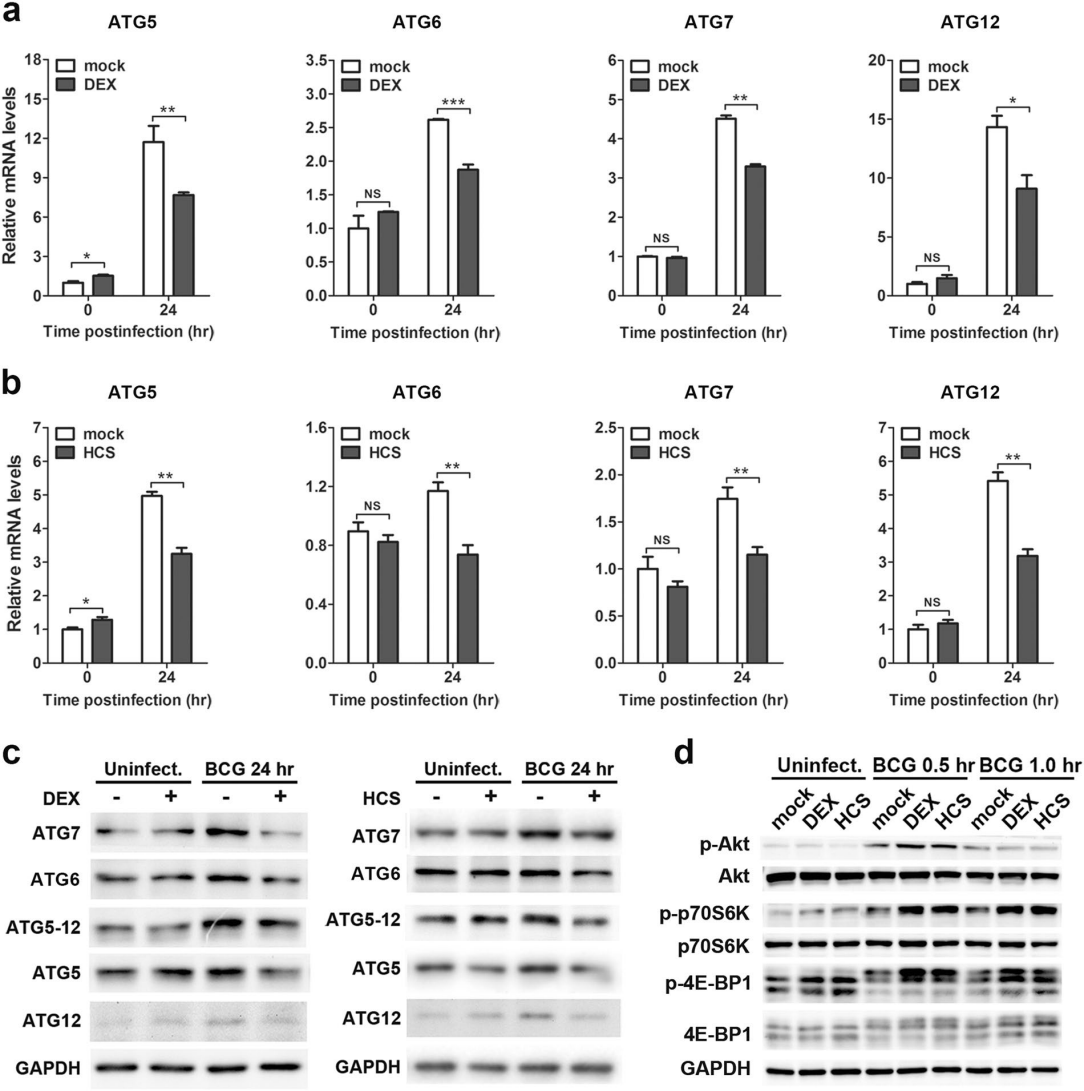
Glucocorticoids inhibit autophagy by suppressing the expression of ATGs and enhancing the activation of the Akt/mTOR pathway. (**a**–**c**) RAW264.7 cells were pretreated with dexamethasone (DEX) (1 μM), hydrocortisone (HCS) (1 μM) or vehicle control ethanol (mock) for 6 hr and then challenged with *M. bovis* BCG (MOI 5) for 24 hr. mRNA and protein levels of ATGs were determined by real-time PCR (**a**,**b**) and Western blot (**c**), respectively. Full-length blots are presented in Supplementary Fig. 10. (**d**) RAW264.7 cells were pretreated with DEX (1 μM), HCS (1 μM) or vehicle control ethanol (mock) for 24 hr and then challenged with *M. bovis* BCG (MOI 5) for the indicated times. Phosphorylation and total protein levels of the indicated genes were determined by Western blot analysis. Full-length blots are presented in Supplementary Fig. 11. For Western blotting, GAPDH was used as the loading control. Data are representative of three independent experiments with similar results. For real-time PCR, the level of each mRNA was normalized to that of β-actin mRNA and is expressed relative to expression in unstimulated cells. Data are shown as the mean ± SEM of three independent experiments. *p < 0.05; **p < 0.01; ***p < 0.001. NS, not significant.

Autophagy is a cascade reaction executed by ATGs^25^. Decreasing or genetically depleting the expression of the key ATGs, such as ATG5, ATG6, ATG7 and ATG12, substantially inhibits the autophagic process^26, 27^. To explore the underlying mechanism for glucocorticoid-mediated suppression of autophagy, we first screened the expression of key ATGs. Notably, dexamethasone down-regulated the mRNA levels of ATGs (ATG5, ATG6, ATG7 and ATG12) in RAW264.7 cells at 24 hpi, while no major alteration was observed for any of these gene in uninfected cells (Fig. 6a). Similarly, hydrocortisone displayed similar effects on the expression of these ATGs at the mRNA level (Fig. 6b). These findings were further verified with Western blot analysis, which showed decreased protein levels of key ATGs after dexamethasone or hydrocortisone treatment (Fig. 6c). Notably, conjugation of ATG5-ATG12 was also decreased after glucocorticoid treatment (Fig. 6c). We next explored the effect of glucocorticoids on the Akt/mTOR pathway, which is recognized as a major regulatory pathway of autophagy. Our results showed that dexamethasone or hydrocortisone treatment increased the phosphorylation level of Akt after *M. bovis* BCG infection (Fig. 6d). Meanwhile, the phosphorylation levels of two mTOR substrates, p70 S6 Kinase (p70S6K) and 4E-BP1, were also upregulated in glucocorticoid-treated macrophages (Fig. 6d). These data indicated that glucocorticoids modulated autophagy by multiple mechanisms, including suppression of ATG expression and enhancement Akt/mTOR pathway activation, in macrophages during mycobacterial infection.

### Glucocorticoids inhibit the clearance of intracellular mycobacteria in macrophages by inhibiting autophagy

**Figure 7:**
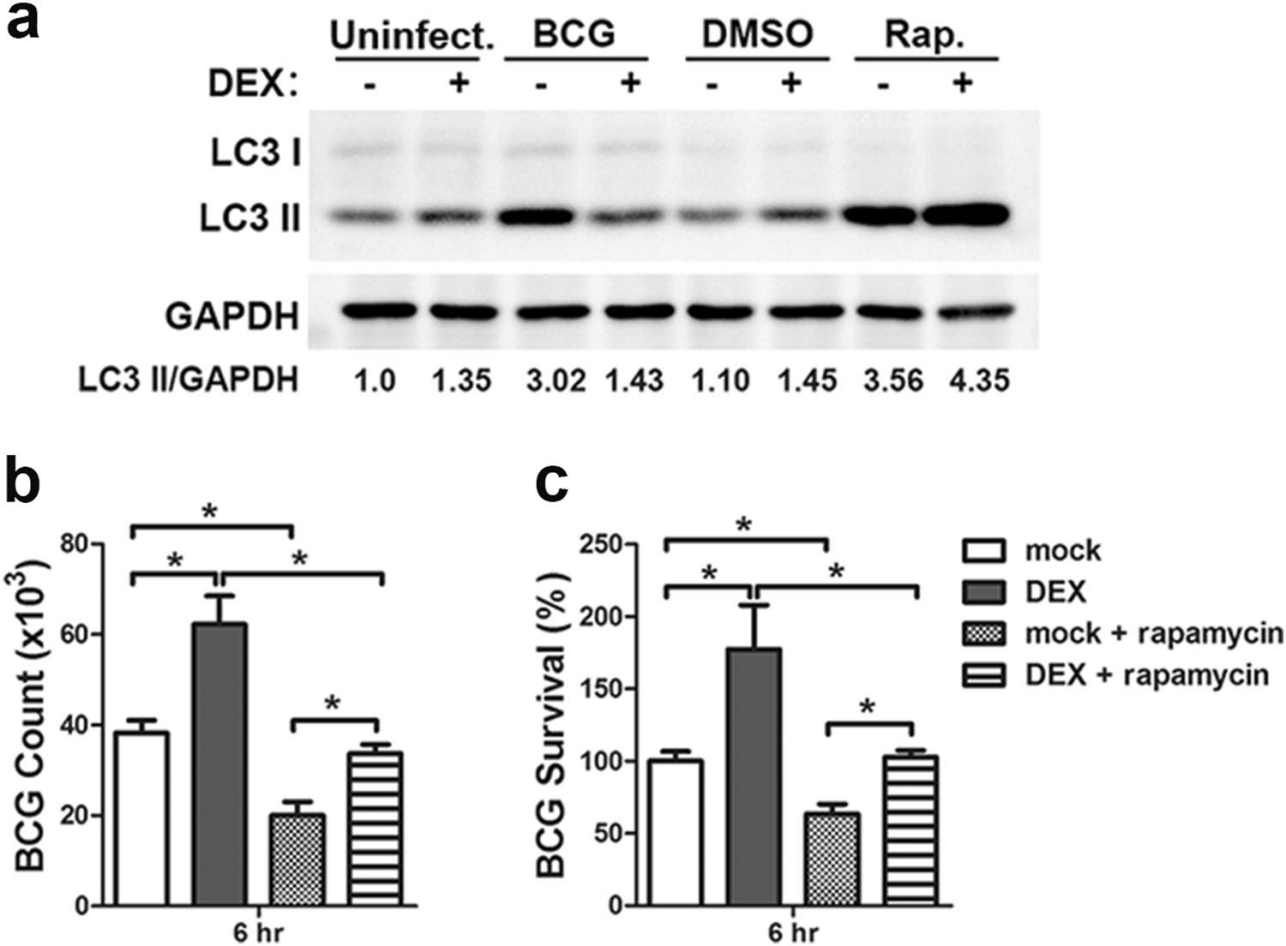
Glucocorticoids promote BCG survival in macrophages by inhibiting autophagy. (**a**) RAW264.7 cells were pretreated with dexamethasone (DEX) (1 μM) or vehicle control ethanol (mock) for 6 hr and then challenged with *M. bovis* BCG (MOI 5) or treated with the vehicle control DMSO or rapamycin (4 μM). LC3 level was detected with Western blot analysis. And GAPDH was used as the loading control. Full-length blots are presented in Supplementary Fig. 12. Data are representative of three independent experiments with similar results. (**b**,**c**) RAW264.7 cells were treated as described in (**a**), and viable bacilli were determined by CFU assays at 6 hpi (**b**). Survival rate was calculated compared with that of mock-treated cells (**c**). Data are shown as the mean ± SEM of three independent experiments. *p < 0.05.

To assess whether glucocorticoids promote mycobacterial survival through autophagy inhibition, we activated autophagy with the agonist rapamycin and determined whether it could restore anti-mycobacterial activity in dexamethasone-treated macrophages. While dexamethasone decreased autophagy in BCG-infected macrophages, rapamycin could effectively induce autophagy in dexamethasone-treated cells (Fig. 7a). Next, we detected clearance of mycobacteria in glucocorticoid-treated macrophages after restoration of autophagy with rapamycin treatment. Consistent with the data above, dexamethasone facilitated mycobacterial survival in RAW264.7 cells. However, after autophagy restoration, viable mycobacteria in dexamethasone-treated macrophages were decreased to levels similar to those in cells without glucocorticoid treatment (Fig. 7b,c). These results suggested that the glucocorticoid-mediated decrease in mycobactericidal activity was due to autophagy inhibition.

## Discussion

Glucocorticoids, potent immunosuppressive agents extensively used in almost all clinical specialties could increase susceptibility to mycobacterial infection after long-term administration^5–7^. Previous reports have focused on glucocorticoid-induced inhibition of adaptive immunity, especially T-cell responses^28–31^. Here, we investigated the effect of glucocorticoids on innate host defense against mycobacterial infection in macrophages. Glucocorticoids impaired antimicrobial NO production, as well as autophagy and maturation of mycobacterial phagosomes in macrophages, thus facilitating the survival of mycobacteria. Activation of TBK1 and expression of several key ATGs were down-regulated by glucocorticoid treatment, while activation of Akt/mTOR pathway was enhanced after glucocorticoid treatment, which account for the inhibition of autophagy. These findings provide novel mechanistic insight into the immunosuppressive activity of glucocorticoids during mycobacterial infection.

Although glucocorticoids are believed to be promising agents to treat exaggerated systemic inflammatory response, such as sepsis, in clinical reports, a systematic review with meta-analysis revealed poor evidence to support the use of steroids in sepsis patients and questioned the alleged benefits^32^. Actually, several experimental studies have identified glucocorticoid intake as an independent risk factor for infections. For example, in a pneumococcal pneumonia model, the inhaled corticosteroid fluticasone reduced alveolar macrophages killing of pneumococci^33^. At the earliest stages of herpes simplex virus-1 infection, glucocorticoids suppressed adaptive antiviral immunity by impairing dendritic cell functions^34^. Here, we provide experimental evidence showing that glucocorticoids impair innate defense and promote mycobacterial survival in both mouse and human macrophages, which is consistent with a recent study showing that the antimicrobial activity of human macrophages was decreased after dexamethasone treatment^35^. According to these findings, it is highly recommended to re-evaluate the benefits and risks before prescribing glucocorticoids to individuals at high-risk of TB. Additionally, essential measures should be taken to prevent reactivation of TB in patients undergoing long-term glucocorticoid therapy.

In previous studies, glucocorticoids have been shown to inhibit innate immune signaling, such as TLR-induced inflammatory responses. Dexamethasone treatment markedly suppressed LPS-mediated induction of inflammatory genes by selective inhibition of p38 MAP kinase^36^. Interferon regulatory factor 3 (IRF3), another signaling branch leading to IFN induction downstream of TLR-3 and TLR-4, was also inhibited by glucocorticoid by targeting TBK1 kinase activation^37^. In our study, we also observed a decrease in the expression of inflammatory cytokines in glucocorticoid treated macrophages after either *M. bovis* BCG or *M. tuberculosis* H37Rv infection. In addition to inflammatory cytokines, NO and autophagy are both important effectors of innate immune responses to mycobacteria^24, 25^. A previous study demonstrated that dexamethasone substantially reduced LPS-induced iNOS expression in murine neonatal microglial cells^38^. In the present study, we found that glucocorticoids inhibited iNOS expression and NO production in mycobacteria-infected macrophages, which is consistent with a previous study demonstrating that hydrocortisone treatment decreased iNOS expression and NO production in a mycobacteria-infected mouse model^39^. Moreover, our results showed that glucocorticoid treatment suppressed autophagy in mycobacteria-challenged macrophages and impaired mycobactericidal activity, which provides new information on the role of glucocorticoid-mediated immunosuppression in innate immunity.

Glucocorticoid-mediated regulation of autophagy has been investigated in other non-infectious models, especially hematological malignancies and musculoskeletal disorders. Glucocorticoids are fundamental drugs commonly used for the treatment of lymphoid leukemia. Autophagy was initiated in lymphoid leukemia cells after dexamethasone treatment and is indispensable for its cytotoxicity^40^. Aberrant expression of ATGs was associated with glucocorticoid resistance in B-cell type acute lymphoblastic leukemia^41^. In osteocytes, dexamethasone triggered autophagy to impair dendritic processes and lead to bone loss^42, 43^. Dexamethasone-induced autophagy regulated the muscle atrophy program^44^. To the best of our knowledge, this work is the first one to investigate glucocorticoid-mediated regulation of autophagy in an infectious setting. Interestingly, our results showed that glucocorticoids inhibited autophagy in mycobacteria-challenged macrophages, in contrast to leukemia cells. Autophagy is initiated and regulated by various extrinsic and intrinsic stimuli/factors, which differ dramatically between distinct cells. This may explain the discrepancy of glucocorticoid-mediated autophagy regulation between mycobacteria-infected macrophages and leukemia cells.

Autophagy is tightly controlled at different stages by distinct factors. The metabolism master regulator mTOR is active under nutrient-rich circumstances and represses autophagy^45^. Although mycobacteria induce autophagy and increase the mTOR kinase activity simultaneously after infection^46^, our study indicated that the present of glucocorticoids further enhanced activation of the Akt/mTOR pathway, which may result in the depression of mycobacteria-mediated autophagy. Rapamycin targets mTOR and inhibits its kinase activity to derepress initiation of autophagy. In this study, we found that glucocorticoids regulated autophagy in macrophages after mycobacterial infection but not rapamycin stimulation. Restoration of autophagy with rapamycin treatment attenuated the promotion of mycobacterial survival mediated by glucocorticoids. These findings indicate that glucocorticoids regulate mycobacteria-induced autophagy, at least partly through enhancement of mTOR signaling.

Glucocorticoids have been reported to inhibit TBK1 phosphorylation and TBK1 kinase activity^37^, which phosphorylates the autophagic adaptor p62 and promotes autophagosome maturation, without affecting the autophagic initiation^23^. In our study, we found that glucocorticoids decreased the activation of TBK1, which may account for the suppression of autophagosome maturation. Autophagy is mediated by a cascade reaction of the ATGs^25^, and decreased expression of key ATGs, such as ATG5, ATG6, ATG7 and ATG12, would substantially inhibit the autophagic process^26, 27^. In the present study, we found that the levels of several key ATGs, including ATG5, ATG6, ATG7 and ATG12, were increased after mycobacterial infection, suggesting that autophagy induced by mycobacteria may be dependent on elevating the expression of key ATGs. However, ATG5, ATG6, ATG7 and ATG12 were suppressed by glucocorticoid treatment, indicating that glucocorticoids inhibit autophagy by decreasing the expression of key ATGs during mycobacterial infection. Thus, glucocorticoids inhibit mycobacteria-induced autophagy in macrophages by multiple mechanisms, leading to the survival of intracellular mycobacteria.

Glucocorticoids function by binding to glucocorticoids receptors (GRs), which induce various inhibitory molecules or repress transcriptional activity of transcription factors to negatively regulate the TLR pathway^47^. Activation of GR was reported to inactivate the transcriptional activity of CCAAT/enhancer binding proteinβ (C/EBPβ) and nuclear factor-erythroid derived 2-like 2 (Nrf2) by recruiting silencing mediator for retinoid and thyroid hormone receptors (SMRT) for histone deacetylation^48^. C/EBPβ and Nrf2 have been shown to modulate the expression of multiple ATGs^49–51^. Thus, glucocorticoids may suppress ATG expression by inhibiting the transcriptional activity of C/EBPβ and Nrf2 during mycobacterial infection, which requires further investigation.

In summary, our results demonstrate a novel mechanism by which glucocorticoids suppress host defense against mycobacterial infection in macrophages. Glucocorticoids repress the activation of TBK1 and attenuate iNOS expression and NO production in macrophages infected with mycobacteria. Moreover, glucocorticoids repress the expression of key ATG, but enhance the activation of the Akt-mTOR pathway during mycobacterial infection, thus decreasing autophagic flux. These changes result in enhancement of intracellular mycobacterial survival. With these findings, we gain new insight into the immunosuppressive activity of glucocorticoids, which may provide useful information about prevention of TB activation.

## Materials and Methods

### Ethics statement

All animal experiments in this study were carried out in accordance with the recommendations in the Guide for the Care and Use of Laboratory Animals of the National Institutes of Health. For all experiments of human primary macrophages, the study was approved by the Ethics Committee of Southern Medical University and conducted in accordance with the Declaration of Helsinki. Written informed consent was obtained from all participants before the commencement of the study. All experimental protocols were reviewed and approved by the Medical Ethics Board and the Biosafety Management Committee of Southern Medical University.

### Reagents

The following reagents were used in this study: Dexamethasone (Sigma-Aldrich, D4902), Hydrocortisone (Sangon Biotech, A610506), Monodansylcadaverine (MDC, Sigma-Aldrich, D4008), Rapamycin (Sigma-Aldrich, V900930), DMSO (Sigma-Aldrich, D2650), Bafilomycin A1 (Santa Cruz, sc-201550), Texas-red (Invitrogen, T-6134), TBK1 inhibitor BX795 (Selleckchem, S1274), Trizol reagent (Invitrogen, 15596-018), Middlebrook 7H9 Broth (BD Difco Laboratories, 271310), Middlebrook 7H10 Agar (BD Difco Laboratories, 262710). The following antibodies were used in this study: LC3 (Novus Biologicals, NB100-2220), GAPDH (ZSGB-BIO, TA-08), CD63 (Santa cruz, sc-15363), ATG5 (Cell Signaling, 12994), ATG6 (Cell Signaling, 3738), ATG7 (Cell Signaling, 8558), ATG12 (Cell Signaling, 4180), β-Tubulin (Cell Signaling, 2128), TBK1/NAK (Cell Signaling, 3013), Phospho-TBK1/NAK (Ser172) (Cell Signaling, 5483), Akt (Cell Signaling, 4691), Phospho-Akt (Ser473) (Cell Signaling, 4060), p70 S6 Kinase (Cell Signaling, 9202), Phospho-p70 S6 Kinase (Thr389) (Cell Signaling, 9206), 4E-BP1 (Cell Signaling, 9644), Phospho-4E-BP1 (Thr37/46) (Cell Signaling, 2855).

### Cell culture

RAW264.7 cells (ATCC; TIB-71) were cultured in DMEM supplemented with 10% fetal bovine serum (FBS) and 100 U/ml penicillin, 100 μg/ml streptomycin. For bone-marrow derived macrophages (BMDMs) preparation, firstly, eight-week-old female C57BL/6 mice were purchased from Southern Medical University Animal Supply Center. Secondly, single-cell suspensions of bone marrow cells from the femurs and tibiae were cultured in DMEM containing 10% FBS, 2 mM L-glutamine, 1 mM sodium pyruvate, 100 U/ml penicillin, 100 μg/ml streptomycin, supplemented with 100 ng/ml GM-CSF. Fresh medium was provided on days 3 and 5 of culture. At days 7 of culture, the isolated macrophages were used for further *in vitro* studies.

### Human monocyte-derived macrophages (MDMs) isolation and culture

Human peripheral blood samples from healthy donors were obtained from Guangzhou Blood Center. Peripheral blood mononuclear cells (PBMCs) were isolated by Ficoll (TBDsciences, Tianjin) density gradient centrifugation. Human monocytes were purified from PBMCs by anti-CD14 microbeads (BD Bioscience) according to the manufacturer’s directions. Monocytes were cultured in RPMI-1640 supplemented with 10% FBS and 100 ng/ml GM-CSF for a week to generate MDMs.

### Bacterial culture and infection

*M. bovis* BCG strain 19015 was purchased from the American Type Culture Collection (ATCC), and mycobacteria were grown in Middlebrook 7H9 broth medium or on 7H10 agar plates supplemented with OADC and cultured in a standard tissue culture incubator as described previously^19, 20, 52^. Mycobacteria were homogenized to generate a single cell suspension, and macrophages were infected with mycobacteria at the indicated multiplicity of infection (MOI).

### Transient Transfection

RAW264.7 cells (at approximately 50% confluence) were transiently transfected with 100 nM TBK1 siRNA vs Negative control (NC) siRNA, using Lipofectamine 2000 (Invitrogen) according to the manufacturer’s instructions. All the above siRNAs were purchased from Ribobio.

### Colony-forming unit (CFU) assays

RAW264.7 cells were infected with *M. bovis* BCG at an MOI of 10. After 1 hr incubation at 37 °C, the infected cells were washed extensively with PBS to remove extracellular mycobacteria, and the infected cells were incubated for indicated time, and then lysed in 1 ml of distilled water. Quantitative culturing was performed using 10-fold serial dilutions. Aliquots of each dilution were inoculated in triplicate on Middlebrook 7H10 agar plates supplemented with OADC. Plates were incubated for 3 weeks before colonies were counted.

### Propidium iodide (PI) staining

RAW264.7 cells were infected with *M. bovis* BCG at an MOI of 10. After 1 hr incubation at 37 °C, the infected cells were washed extensively with PBS to remove extracellular mycobacteria, and the infected cells were incubated for indicated time. Cells were collected and stained with PI (Sangon Biotech, E607306) according to the manufacturer’s instructions, and measured by flow cytometry.

### Western blot analysis

Western blot analysis was performed as described previously^53, 54^. Briefly, the whole-cell extract was resolved by SDS-polyacrylamide gel electrophoresis and transferred to polyvinylidene fluoride membranes. The membranes were blocked in 5% bovine serum albumin and then incubated with diluted primary antibodies at 4 °C overnight. Western blot analysis was developed using horseradish peroxidase-conjugated secondary antibody, followed by detection with enhanced chemiluminescence.

### Confocal microscopy

Confocal microscopy was performed as described previously^19^. For immunofluorescence experiments, cells were grown on glass coverslips and treated as indicated, followed by fixation, permeabilization and blocking. Samples were incubated with primary antibody at 4 °C overnight, and then with secondary antibody for 1 hr at room temperature before mounting. In fluorescence experiments, RAW264.7 GFP-LC3 cells were treated as indicated, followed by fixation, permeabilization. Nuclei were labeled with 4,6-diamidino-2-phenylindole (DAPI) staining. MDC staining was performed by adding MDC (50 μM) to cells and incubating at 37 °C for 30 min before fixation. Coverslips were mounted with ProLong Gold antifade reagent (Invitrogen) and visualized using Olympus confocal microscope (FV10-ASW).

### RNA isolation and real time-PCR

Total RNA was isolated with Trizol reagent as described before^19, 55, 56^ and cDNA was synthesized with TransScript One-Step gDNA Removal and cDNA Synthesis SuperMix (TransGen Biotech, AT311-03). TransStart Top Green qPCR SuperMix and an Eppendorf Mastercycler ep realplex^4^ Real-Time PCR system (Eppendorf) were used for quantitative RT-PCR analysis. β-actin was used as housekeeping gene for data normalization. The primer sequences used for PCR were: β-actin, 5′-GATTACTGCTCTGGCTCCTAGC-3′ (forward) and 5′-GACTCATCGTACTCCTGCTTGC-3′ (reverse); ATG5, 5′-TGCTGAAGGCTGTAGGAGAC-3′ (forward) and 5′-CGAGGCCACCAGTTTAAGGA-3′ (reverse); ATG6, 5′-CAGGAGGAAGAGGCTAACTCAG-3′ (forward) and 5′-GTGACCTTGAGTCTCCGGCT-3′ (reverse); ATG7, 5′-GCAC GAACTGACCCAGAAGA-3′ (forward) and 5′-GTTGGTGTTGTGCAGGGTTC-3′ (reverse); ATG12, 5′-TGCTGAAGGCTGTAGGAGAC-3′ (forward) and 5′-CGAGGCCACCAGTTTAAGGA-3′ (reverse); iNOS, 5′-TCCTCACTGGGACAGCACAGA ATG-3′ (forward) and 5′-GTGTCATGCAAAATCTCTCCACTGCC-3′ (reverse); TBK1, 5′-TGTTCTAGAGGAGCCGTCCA-3′ (forward) and 5′-GGTGCACTATGCCGTTCTCT-3′ (reverse).

### Griess assay

RAW264.7 cells were pretreated with dexamethasone, hydrocortisone, BX795, or transfection with Negative control/TBK1 siRNA, and then the supernatant of each sample was collected at 0, 6 and 24 hr after *M. bovis* BCG challenge. NO levels were determined by measuring its stable end product, nitrite, using a Griess reagent (Sigma, St. Louis, Missouri). One hundred microliter of supernatant was added to an equal volume of Griess reagent in duplicate on a 96-well plate and incubated at room temperature for 15 min. Absorbance (540 nm) was measured and nitrite concentrations were estimated using a standard nitrite curve. Results were expressed as the mean micromoles of nitrite per sample ± SEM.

### Statistical analysis

Statistical analysis was performed using GraphPad Prism 5.0 (GraphPad Software, San Diego, CA). Real-time PCR and CFU data are shown as the mean ± SEM. Data between two groups were compared by using Student’s *t*-test. Differences were considered statistically significant with a *p* value less than 0.05.

## Electronic supplementary material

Supplementary Information
