# Supplementary material for: Glucocorticoids Suppress Antimicrobial Autophagy and Nitric Oxide Production and Facilitate Mycobacterial Survival in Macrophages
Source: Sci Rep. 2017 Apr 20;7:982. doi: 10.1038/s41598-017-01174-9 (PMC5430514; doi:10.1038/s41598-017-01174-9)

Supplementary Information

Glucocorticoids Suppress Antimicrobial Autophagy and Nitric Oxide Production and Facilitate Mycobacterial Survival in Macrophages

Jinli Wang[1], Ruining Wang[1], Hui Wang[1], Xiaofan Yang[1], Jiahui Yang[1], Wenjing Xiong[1], Qian Wen[1], and Li Ma[1,*]

1. Institute of Molecular Immunology, School of Laboratory Medicine and Biotechnology, Southern Medical University, Guangzhou 510515, China

* Correspondence:

Li Ma, M.D., Ph.D.

Institute of Molecular Immunology, School of Laboratory Medicine and Biotechnology, Southern Medical University, Guangzhou 510515, China

Tel: 86-20-6164-8322, Fax: 86-20-6164-8322

Email: maryhmz@126.com

Supplementary Figures and Figure Legends

Supplementary Figure 1

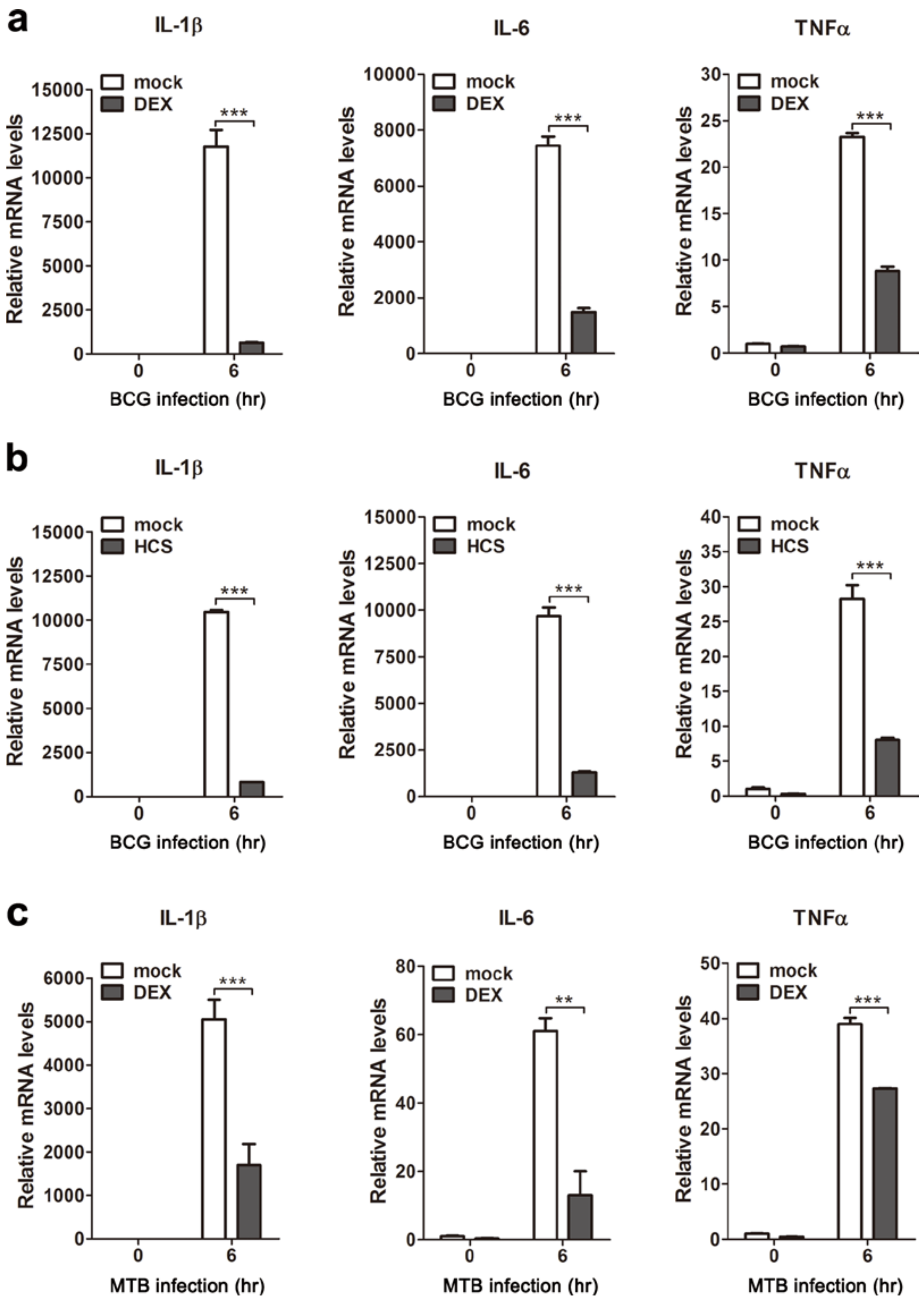

Figure S1. Glucocorticoids inhibit inflammatory cytokines expression in mycobacteria-challenged macrophages. (**a** and **b**) RAW264.7 cells were pretreated

with vehicle control ethanol, dexamethasone (DEX, 1 μM) (**a**) or hydrocortisone (HCS, 1 μM) (**b**) for 24 hr and then challenged with *M. bovis* BCG (MOI 5) for 6 hr. (**c**) RAW264.7 cells were pretreated with vehicle control ethanol or dexamethasone (DEX, 1 μM) for 24 hr and then challenged with MTB (MOI 5) for 6 hr. mRNA expression levels of IL-1β, IL-6 and TNFα were detected with real-time PCR. The level of each mRNA was normalized to that of β-actin mRNA and is expressed relative to expression in unstimulated cells. Data are shown as mean±SEM of three independent experiments. Statistical significance was determined using the Student *t* test. **, p<0.01; ***, p<0.001.

Supplementary Figure 2

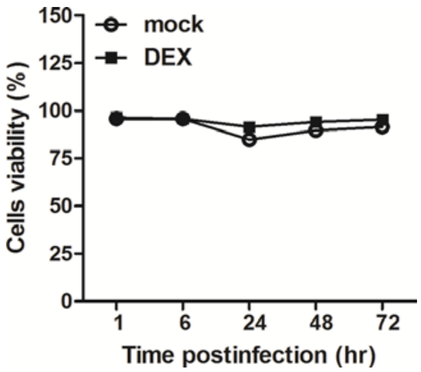

Figure S2. Effects of glucocorticoid on cell viability after mycobacterial infection. RAW264.7 cells were pretreated with vehicle control ethanol or DEX (1 μM) for 24 hr and then challenged with *M. bovis* BCG (MOI 10) for 1 hr. The infected cells were washed extensively with PBS to remove extracellular mycobacteria. Cells were collected after incubated for the indicated time and stained with propidium iodide (PI) to test cell viability. The percentage of cells viability was shown.

Supplementary Figure 3

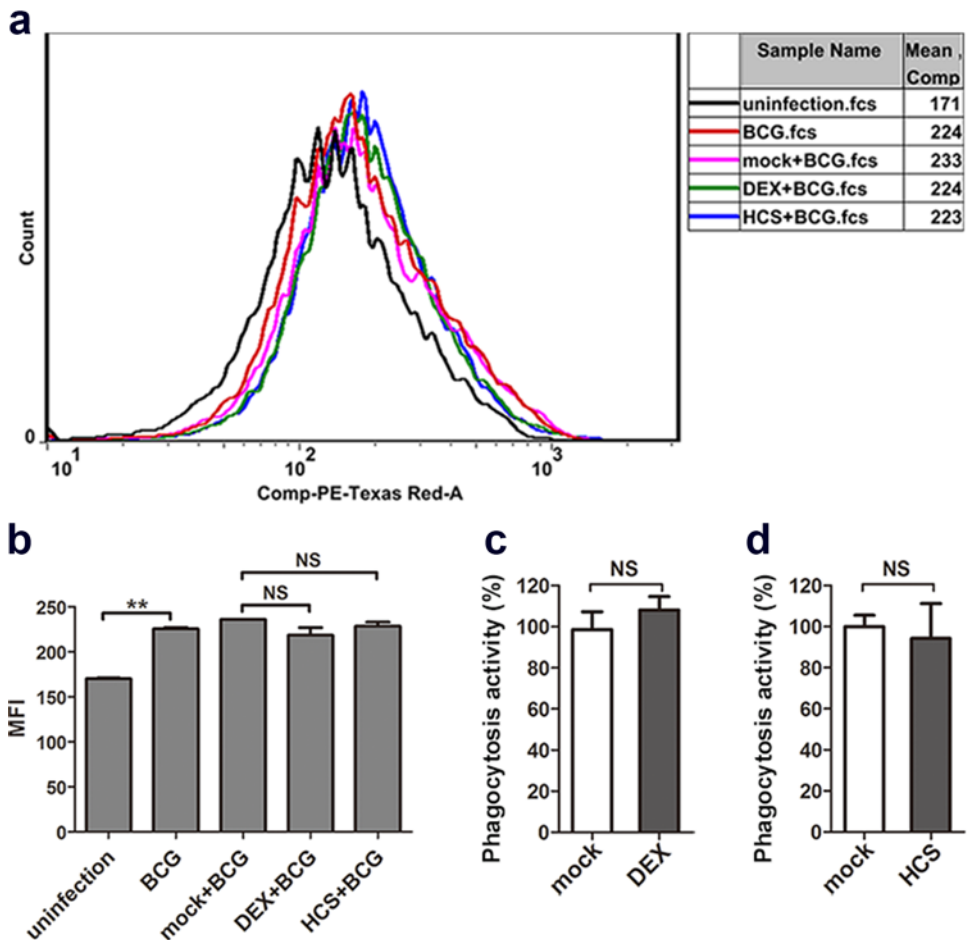

Figure S3. Glucocorticoids have no major effect on phagocytosis of mycobacteria in macrophages. RAW264.7 cells were pretreated with vehicle control ethanol, DEX (1 μM) or HCS (1 μM) for 24 hr and then challenged with Texas-Red-labeled *M. bovis* BCG (MOI 10) for 1 hr. Phagocytosis of *M. bovis* BCG was determined by flow cytometry (**a**). Mean fluorescence intensity (MFI) was calculated (**b**). Uptake of *M. bovis* BCG was determined by CFU assays (**c**,**d**). The phagocytosis ability of glucocorticoid-treated cells was compared with that of mock-treated cells. Data are shown as the mean ± SEM of three independent experiments. Statistical significance was determined using the Student's *t* test. **, $p<0.01$. NS, not significant.

Supplementary Figure 4. The full-length blots is the display of cropped blots from Figure 1d.

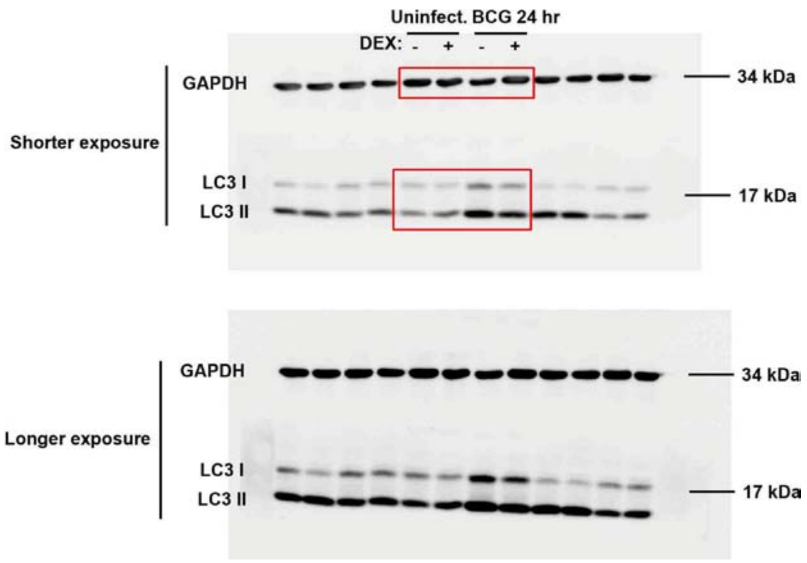

Supplementary Figure 5. The full-length blots is the display of cropped blots from Figure 1e.

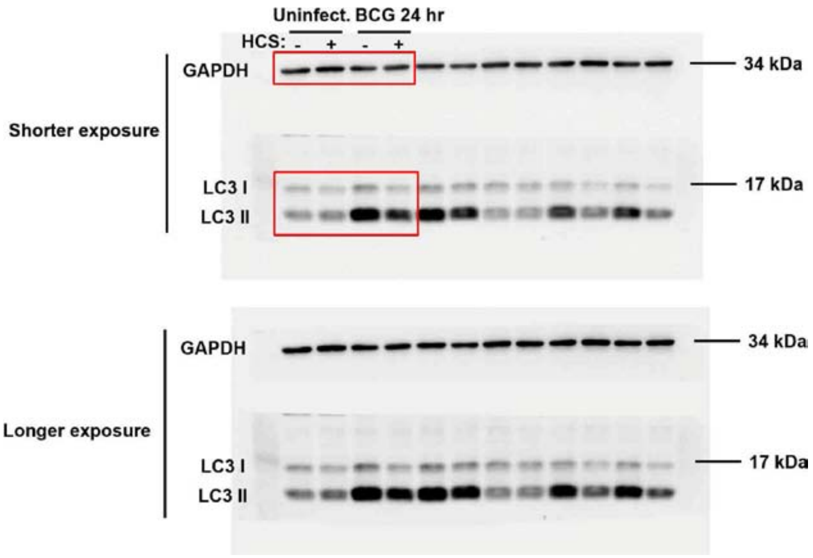

Supplementary Figure 6. The full-length blots is the display of cropped blots from Figure 1f.

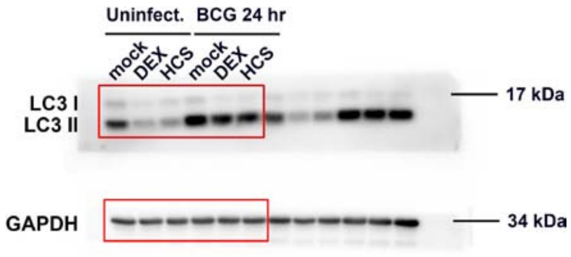

Supplementary Figure 7. The full-length blots is the display of cropped blots from Figure 1g.

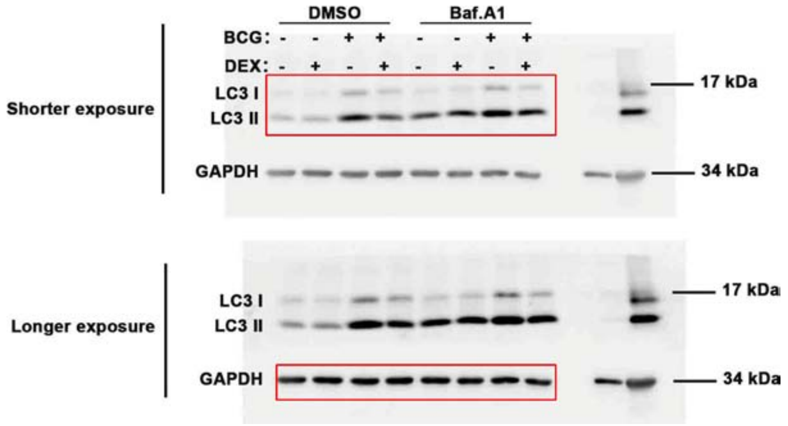

Supplementary Figure 8. The full-length blots is the display of cropped blots from Figure 5a.

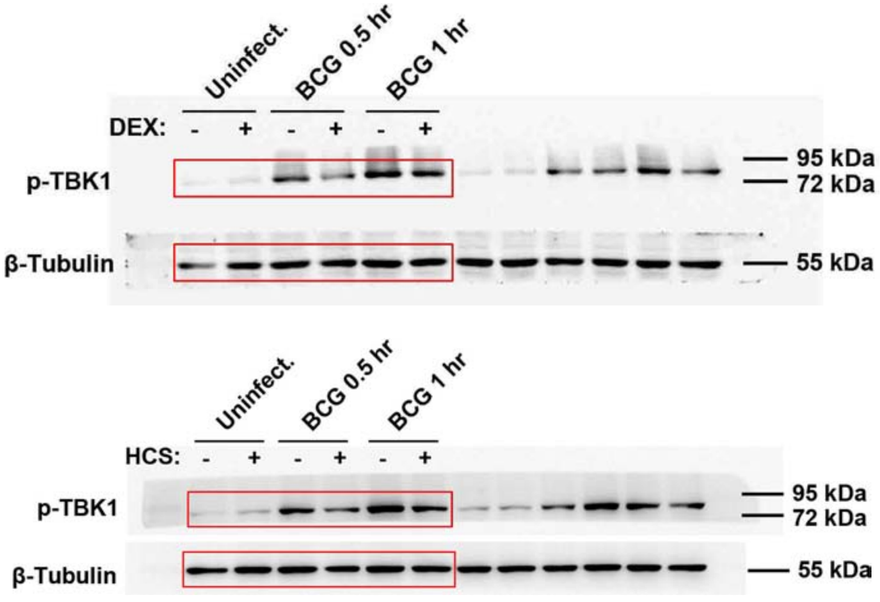

Supplementary Figure 9. The full-length blots is the display of cropped blots from Figure 5b.

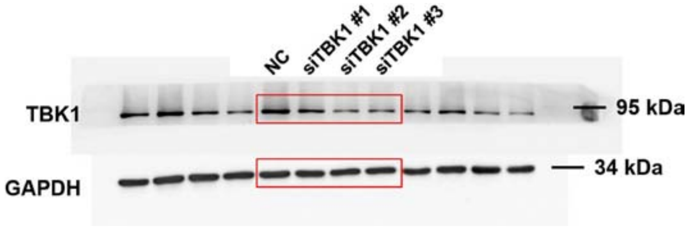

Supplementary Figure 10. The full-length blots is the display of cropped blots from Figure 6c. The samples for detecting ATGs and GAPDH were derived from the same experiment and that blots were processed in parallel.

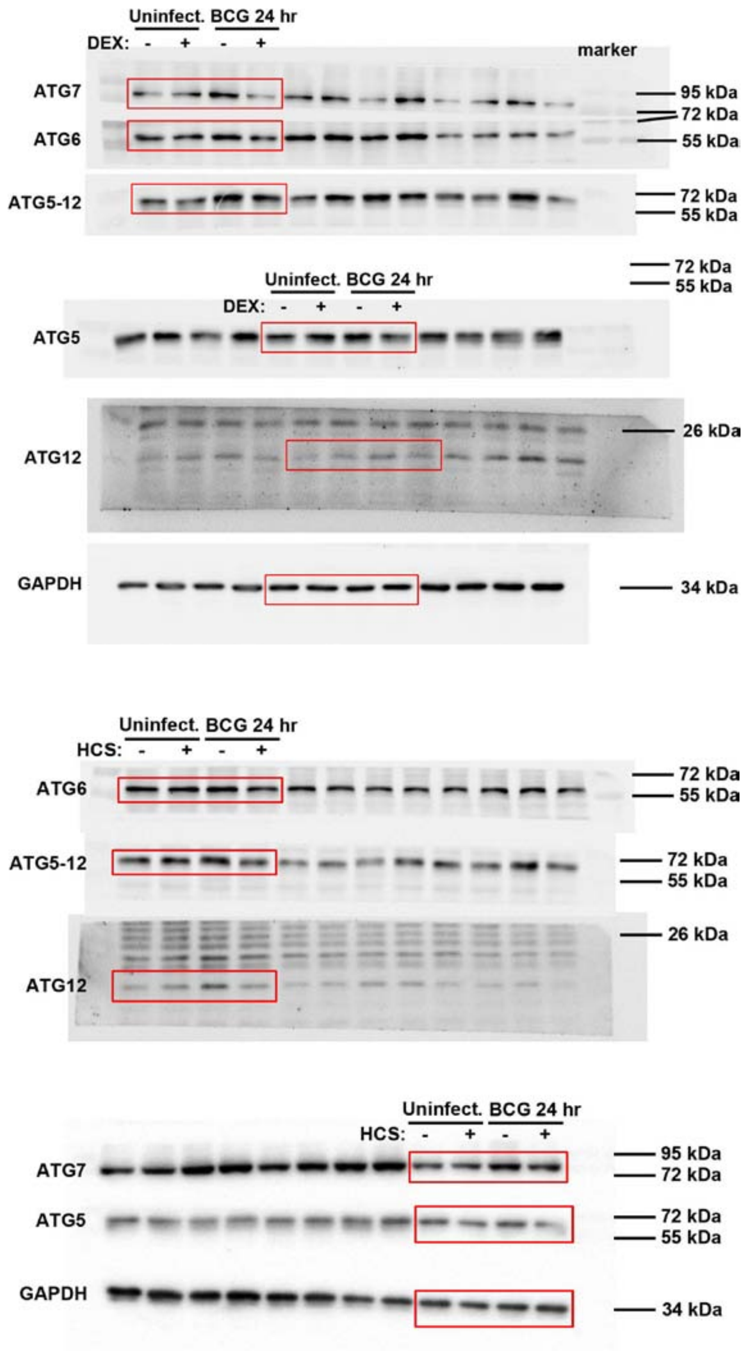

Supplementary Figure 11. The full-length blots is the display of cropped blots from Figure 6d.

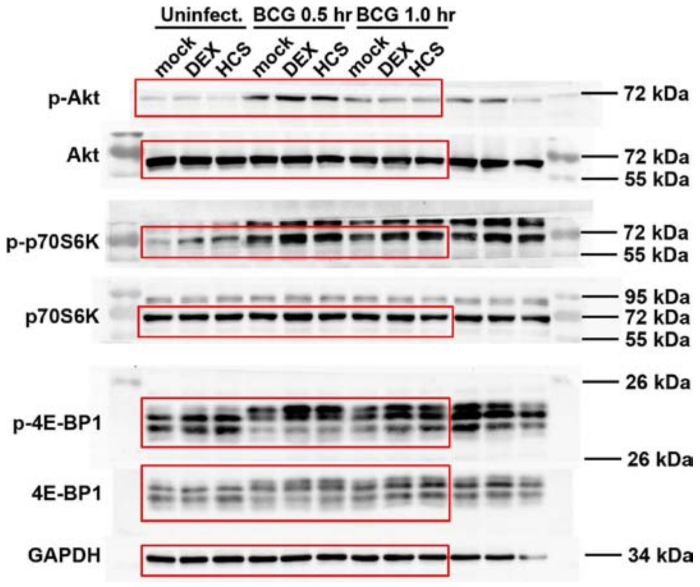

Supplementary Figure 12. The full-length blots is the display of cropped blots from Figure 7a.

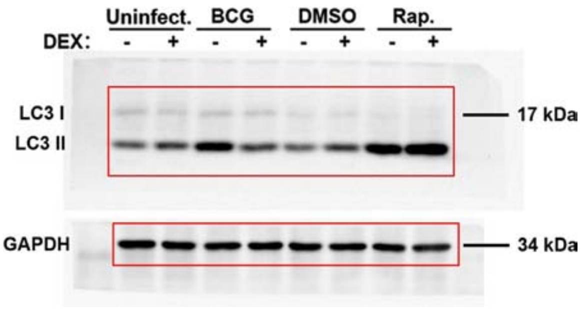

Supplement: Supplementary file 1 — Supplementary Information [file 41598_2017_1174_MOESM1_ESM.pdf]
